# sFlt Multivalent Conjugates Inhibit Angiogenesis and Improve Half-Life *In Vivo*

**DOI:** 10.1371/journal.pone.0155990

**Published:** 2016-06-03

**Authors:** Eda I. Altiok, Shane Browne, Emily Khuc, Elizabeth P. Moran, Fangfang Qiu, Kelu Zhou, Jorge L. Santiago-Ortiz, Jian-xing Ma, Matilda F. Chan, Kevin E. Healy

**Affiliations:** 1 Department of Bioengineering, University of California, Berkeley, California, United States of America; 2 Centre for Research in Medical Devices (CÚRAM), National University of Ireland Galway, Galway, Ireland; 3 Department of Ophthalmology, University of California at San Francisco, San Francisco, California, United States of America; 4 Department of Physiology, Harold Hamm Diabetes Center, University of Oklahoma Health Sciences Center, Oklahoma City, Oklahoma, United States of America; 5 Department of Chemical and Biomolecular Engineering, University of California, Berkeley, California, United States of America; 6 Department of Materials Science and Engineering, University of California, Berkeley, California, United States of America; University of Melbourne, AUSTRALIA

## Abstract

Current anti-VEGF drugs for patients with diabetic retinopathy suffer from short residence time in the vitreous of the eye. In order to maintain biologically effective doses of drug for inhibiting retinal neovascularization, patients are required to receive regular monthly injections of drug, which often results in low patient compliance and progression of the disease. To improve the intravitreal residence time of anti-VEGF drugs, we have synthesized multivalent bioconjugates of an anti-VEGF protein, soluble fms-like tyrosine kinase-1 (**sFlt**) that is covalently grafted to chains of hyaluronic acid (HyA), conjugates that are termed mvsFlt. Using a mouse corneal angiogenesis assay, we demonstrate that covalent conjugation to HyA chains does not decrease the bioactivity of sFlt and that mvsFlt is equivalent to sFlt at inhibiting corneal angiogenesis. In a rat vitreous model, we observed that mvsFlt had significantly increased intravitreal residence time compared to the unconjugated sFlt after 2 days. The calculated intravitreal half-lives for sFlt and mvsFlt were 3.3 and 35 hours, respectively. Furthermore, we show that mvsFlt is more effective than the unconjugated form at inhibiting retinal neovascularization in an oxygen-induced retinopathy model, an effect that is most likely due to the longer half-life of mvsFlt in the vitreous. Taken together, our results indicate that conjugation of sFlt to HyA does not affect its affinity for VEGF and this conjugation significantly improves drug half-life. These *in vivo* results suggest that our strategy of multivalent conjugation could substantially improve upon drug half-life, and thus the efficacy of currently available drugs that are used in diseases such as diabetic retinopathy, thereby improving patient quality of life.

## Introduction

Diabetes affects 285 million adults worldwide, which accounts for 6.4% of the world’s population. This number is expected to increase to 439 million people by 2030 [1]. In the United States alone, 25.8 million people have diabetes and it is estimated that another 57 million people are considered pre-diabetic as a result of abnormally high blood glucose levels resulting from poor diet. Diabetic retinopathy is one of the most debilitating consequences of diabetes and is currently the leading cause of blindness among individuals aged 20–74 [2–4].

In the development of diabetic retinopathy, hypoxic neurons upregulate growth factors that drive retinal angiogenesis, a process primarily mediated by vascular endothelial growth factor (VEGF) [5–7]. Newly formed blood vessels have loose cell-cell junctions, causing macular edema and pooling of blood, which leads to areas of vision loss. Anti-VEGF therapeutics such as Lucentis (ranibizumab, Genentech, 48 kDa humanized antibody fragment), Avastin (bevacizumab, Genentech, a 150 kDa humanized antibody) and Eylea (aflibercept, Regeneron, a 110 kDa VEGF-receptor fusion protein) are the clinically used drugs for treating diabetic retinopathy in the clinic. However, these therapeutics suffer from short drug half-lives as a result of small molecular size and single injections of these drugs provide limited therapeutic effect over a finite period of time [8]. Consequently, low levels of patient compliance significantly limit the effect of these drugs to maintain visual acuity [9,10]. Therefore, the development of new drug delivery approaches is necessary to facilitate increased drug residence time within the vitreous. Such approaches will help to reduce the frequency at which patients are required to receive injections thereby improving quality of life as well as long term visual potential.

We have developed a novel multivalent drug technology platform that allows for the long-term delivery of anti-VEGF drugs into the vitreous. Using a naturally occurring biopolymer, hyaluronic acid (HyA) we synthesized drug multivalent conjugates by chemically conjugating sFlt (soluble fms-like tyrosine kinase-1 [11]), an anti-VEGF protein, to the biopolymer chain thereby significantly increasing the overall drug size. We have shown previously that these anti-VEGF multivalent conjugates, mvsFlt, maintain their ability to bind VEGF and inhibit endothelial cell proliferation, migration and tube formation *in vitro*. Furthermore, conjugation of sFlt to HyA significantly enhanced retention within *in vitro* models of the vitreous and protected sFlt from protease degradation by matrix metalloproteinase-7, an enzyme that specifically degrades sFlt [12]. The aim of this study was therefore to evaluate the *in vivo* activity of the mvsFlt conjugate. We used a corneal angiogenesis assay as an initial screen for examining the effect of HyA conjugation on sFlt bioactivity and then investigated the half-life of mvsFlt in the rat vitreous in order to determine how HyA conjugation affected residence time of sFlt. Finally, we employed a rat oxygen-induced retinopathy (OIR) model to examine both bioactivity and the effect of increased mvsFlt half-life on retinal angiogenesis. The results from this study suggest our conjugation technology may have far reaching impacts on the development of pharmaceuticals with longer half-lives in the vitreous not only for treating diabetic retinopathy but also for retinal neovascular diseases such as wet age-related macular degeneration (wet-AMD).

## Materials and Methods

### 2.1. Expression of Soluble Flt-1 Receptor

The sFlt sequence for the first 3 Ig-like extracellular domains of sFlt-1 was cloned as previously described [13] into the pFastBac1 plasmid (Life Technologies) and then transformed into DH10Bac *E*.*coli*, which were plated on triple antibiotic plates containing kanamycin (50 μg/mL, Sigma Aldrich), gentamicin (7 μg/mL, Sigma Aldrich), tetracycline (10 μg/mL, Sigma Aldrich), IPTG (40 μg/mL, Sigma Aldrich) and Bluo-gal (100 μg/mL, Thermo Fisher Scientific). The sFlt gene-containing bacmid was isolated from DH10Bac *E*.*coli* (Life Technologies) and transfected into SF9 insect cells for virus production (provided by the Tissue Culture Facility, UC Berkeley). Virus was then used to infect High Five insect cells (provided by the Tissue Culture Facility, UC Berkeley) to induce sFlt protein expression. After 3 days, protein was purified from the supernatant using Ni-NTA agarose beads (Qiagen Laboratories). Recombinant sFlt was eluted from the Ni-NTA beads using an imidazole (Sigma Aldrich) gradient and then concentrated and buffer exchanged with 10% glycerol/PBS using Amicon Ultra-15mL Centrifugal devices (EMD Millipore). The protein solution was sterile filtered and the concentration was determined by BCA assay (Thermo Fisher Scientific). The molecular weight of sFlt was determined from a 4–20% gradient SDS-PAGE gel and estimated to be 50 kDa [12].

### 2.2 mvsFlt Conjugate Synthesis

**Fig 1:**
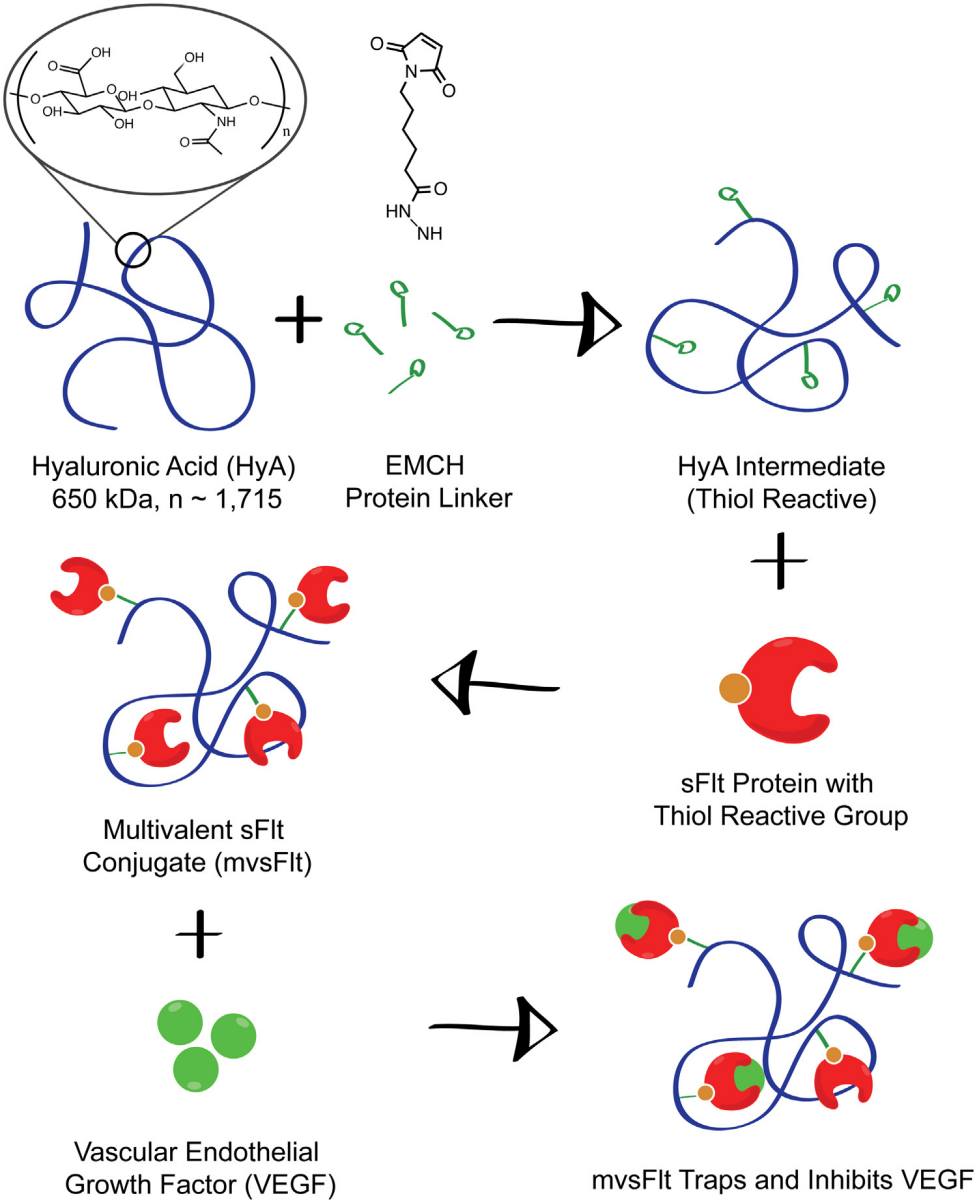
Synthesis of mvsFlt schematic. mvsFlt bioconjugates were synthesized using a 3-step reaction in which HyA was reacted with EDC and EMCH to create a thiol reactive HyA-EMCH intermediate. sFlt was then treated with 2-iminothiolane and reacted with the HyA-EMCH intermediate for the synthesis of the final mvsFlt bioconjugate.

Conjugation of sFlt to HyA was carried out according to the schematic in Fig 1, and as described previously ([12,14–16]. To make thiol-reactive HyA intermediates, 3,3′-*N*-(ε-maleimidocaproic acid) hydrazide (EMCH, Pierce, 1.2 mg/mL), 1-hydroxybenzotriazole hydrate (HOBt, Sigma, 0.3mg/mL) and 1-ethyl-3-(3-dimethylaminopropyl) carbodiimide hydrochloride (EDC, Pierce, 10 mg/mL) were added to a 3 mg/ml solution of 650 kDa HyA (Lifecore Biotechnology) in 0.1 M 2-(*N*-morpholino) ethanesulphonic acid (MES) (Sigma) buffer (pH 6.5) and allowed to react at 4°C for 4 h. The solution was then dialyzed into pH 7.0 phosphate-buffered saline (PBS) containing 10% glycerol. Recombinant sFlt was treated with 2-iminothiolane at 10 molar excess to create thiol groups for conjugation to the maleimide group on EMCH. Activated HyA-EMCH was then added to sFlt at a 1:10 molar ratio (HyA to sFlt) and allowed to react at 4°C overnight to synthesize the final mvsFlt conjugate. The mvsFlt conjugate was dialyzed with 100 kDa molecular weight cut-off (MWCO) Float-A-Lyzer G2 (Spectrum Labs) in pH 7.0 PBS exhaustively to remove unreacted sFlt. The concentration of mvsFlt was measured using a BCA assay.

### 2.3 Corneal Angiogenesis Assay

All experiments were performed with a total of 32 wild-type 7 to 12-week old male and female littermate FVB/n mice. Mice were maintained under pathogen-free conditions in the UCSF barrier facility and experiments conducted in accordance with procedures approved by the UCSF Institutional Animal Care and Use Committee (IACUC). All experiments were approved by the UCSF IACUC prior to work. Mice were anesthetized by isofluorane inhalation (Abbott Laboratories, Abbott Park, IL), 10mg/kg carprofren (Sigma, St. Louis, MO), and by topical application of 0.5% Proparacaine (Bausch & Lomb, Rochester, NY) placed on the cornea. An alkaline burn was created by applying filter paper 2.5 mm in diameter soaked in 0.1N NaOH (Sigma Aldrich) for 30 seconds to the central cornea followed by rinsing with 250 μL of PBS. After the chemical burn treatment, topical 0.5% proparacaine was added to the cornea for anesthesia. Mice were administered 5 μL subconjunctival injections with sFlt (150 μg/ml), mvsFlt (150 μg/ml), or PBS at day 1 and day 3 after burn. Ten days after treatment, eyes were enucleated and the corneas were dissected and fixed in 4% paraformaldehyde overnight at 4°C. Corneas were blocked with 3% BSA and stained with DAPI, rabbit anti-mouse CD31 primary antibody (Santa Cruz Biotechnology) and goat anti-rabbit Alexa Fluor 488 secondary antibody (Life Technologies) for visualization and quantification of blood vessels. Corneas were cut into quadrants and flat-mounted onto glass slides using Fluoromount Mounting Medium (Sigma Aldrich). Imaging was carried out with an automated slide scanner, Zeiss Axioscan Z1 (Zeiss Instruments). Corneal blood vessel coverage was quantified using NIH ImageJ software by comparing the total cornea area to the corneal vascularized area.

### 2.4 Determination of mvsFlt Intravitreal Residence Time

All residence time experiments were performed on 8-week old Brown Norway rats (24 rats total with 12 in each group) obtained from Charles River Laboratories and treated in accordance with protocols approved by the Institutional Animal Care and Use Committee at UC Berkeley. All experiments were approved by the UC Berkeley IACUC prior to work. Rats were anesthetized using a mixture of ketamine and xylazine (50 mg and 10 mg/kg body weight, respectively) for the surgical procedure. Eyes were injected intravitreally 1 mm behind the limbus with 5 μL of PBS, sFlt or mvsFlt at 1 mg/mL using a 30-gauge Hamilton syringe and monitored daily for signs of inflammation. This concentration was selected to maximize fluorescence in the vitreous and remain in the detection limit of the fluorometer after 48 hours. Rats were sacrificed with CO_2_ asphyxiation in groups at 0, 4, 12, 24 and 48 hours post injection and eyes were immediately enucleated and placed on dry ice. Frozen vitreous was then extracted from the eye and immersed in 100 μL of RIPA buffer. After shaking on ice for 2 hours, each vitreous sample was homogenized with a Tissue Tearor (Bio Spec Products, Inc.) and the fluorescence measured using a fluorometer (Molecular Devices). Quantification was carried out by normalizing the fluorescence of vitreous samples to the 0 hour vitreous fluorescence readings within their respective group. The half-lives of sFlt and mvsFlt were calculated according to Eq 1:

$$C_{t}=C_{0}e^{-kt}$$ (1)

Where *C*_*t*_ is the concentration at time *t*, *C*_0_ is the initial concentration and *k* is the elimination constant given by Eq 2:

k=log(2)t1/2 (2)

Where *t*_1/2_ is the drug half-life. The values used for calculating *t*_1/2_ were based on data from the 48-hour time point.

### 2.5 OIR Rat Angiogenesis Model

Pregnant Brown Norway rats were obtained from Charles River Laboratories. All the animal experiments were performed in compliance with the ARVO statement for the Use of Animals in Ophthalmic and Vision Research and approved by the University of Oklahoma Institutional Animal Care and Use Committee. Newborn pups were assigned to PBS, sFlt or mvsFlt treatment groups (6 rat pups in each group). Light was cycled on a 12 hour on, 12 hour off schedule and room temperature was maintained at approximately 21C. Rat pups were exposed to hyperoxia (75% O_2_) from postnatal day 7 (P7) to P12. The oxygen-treated rats were housed in an incubator connected to an Oxycler Model A4 (Redfield, NY) with oxygen and nitrogen, allowing for adjustment of oxygen concentration to 75%±2%. The rats were placed in the oxygen chamber with enough food and water to sustain them for 5 days. On P12, the animals were returned to room air and administered intravitreal injections with 2 μL per eye of PBS, sFlt or mvsFlt at 150 μg/mL. Rats at P17 were anesthetized and perfused with high-molecular weight FITC-dextran (2 MDa; Sigma-Aldrich, St. Louis MO) as described by Smith et al [17]. Retinas were dissected and flat-mounted and the vasculature was imaged using a fluorescence microscope (CKX41; Olympus). Vascular coverage at P17 was quantified using NIH ImageJ by comparing the total retinal area to the area of vascularization.

### 2.6 Statistical Analysis

Values are expressed as means ± standard deviations (SD). Statistical analysis was performed with two-tailed *t-*tests to compare mean values. One-way (with Tukey *post-hoc* analysis) and two-way ANOVA (with Bonferroni posttest) were also used to compare treatment groups in the quantitative measurements where appropriate (Prism, GraphPad Software). A P-value of less than 0.05 was considered to be statistically significant.

## Results

### 3.1 sFlt and mvsFlt equally inhibit corneal angiogenesis

**Fig 2:**
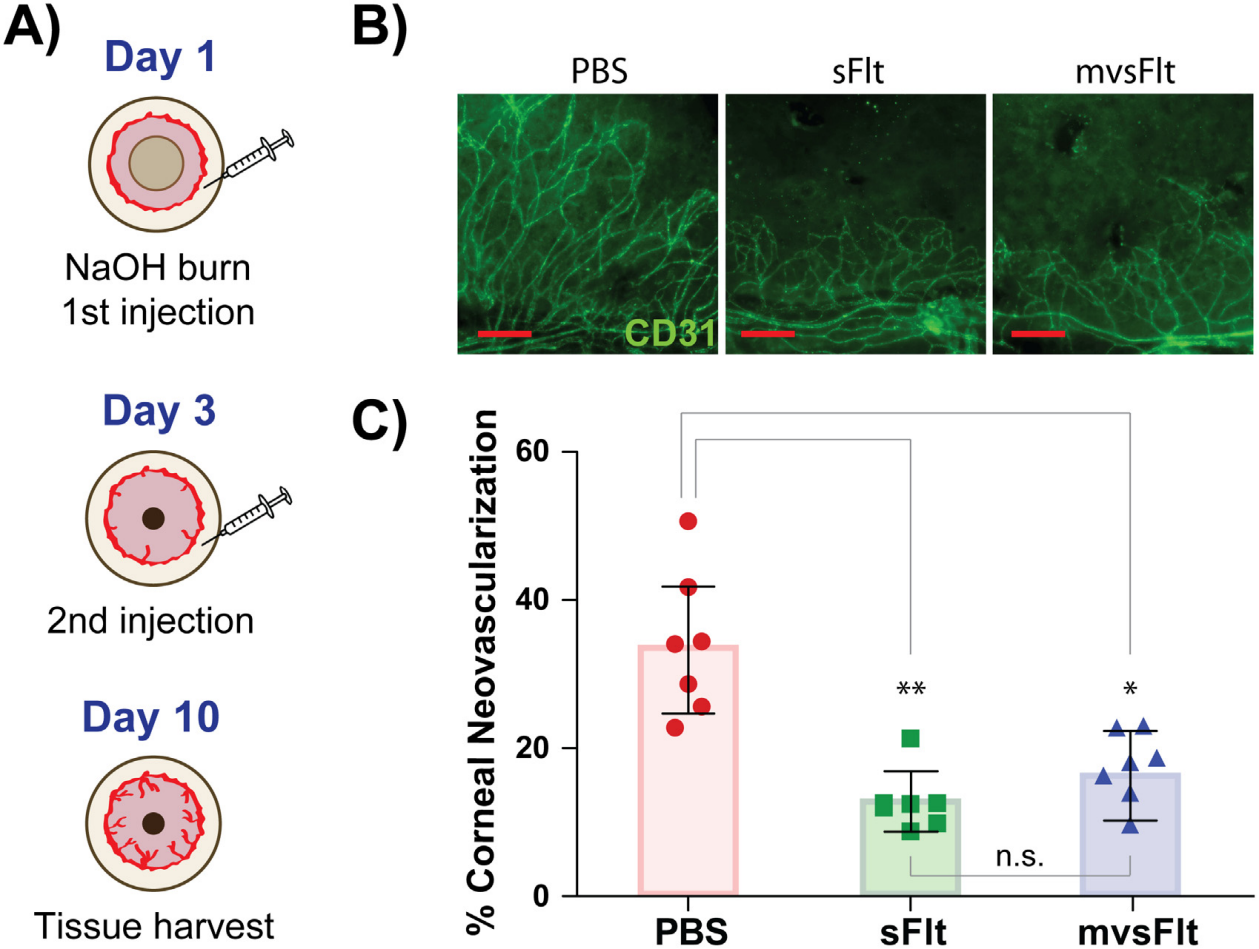
sFlt and mvsFlt equally inhibit corneal angiogenesis. A) Schematic depicting methods utilized for carrying out corneal burn model. Mice were treated twice with 5μl of PBS, sFlt or mvsFlt at day 1 and 3 following the chemical burn. B) Representative images of eyes treated with PBS, sFlt and mvsFlt. CD31 positive (green) staining of corneal blood vessels. C) Quantification of corneal angiogenesis at day 10 following treatment. One-way ANOVA gives p value **<0.01 (n.s.- not significant; *p<0.05; **p<0.01). Scale bars correspond to 20 μm.

The chemical injury-based corneal angiogenesis model was used to determine whether conjugation of sFlt to HyA reduced the bioactivity of mvsFlt to in comparison to sFlt *in vivo*. Ten days-post corneal injury, all mice treated with sFlt and mvsFlt displayed similar inhibitory profiles of corneal angiogenesis (Fig 2). Corneas treated with PBS had 28.8±11.5% blood vessel coverage in contrast to corneas treated with sFlt and mvsFlt, which had 12.8±3.8% and 15.8±7.1% vascular coverage, respectively.

### 3.2 mvsFlt has significantly longer residence time in vitreous

**Fig 3:**
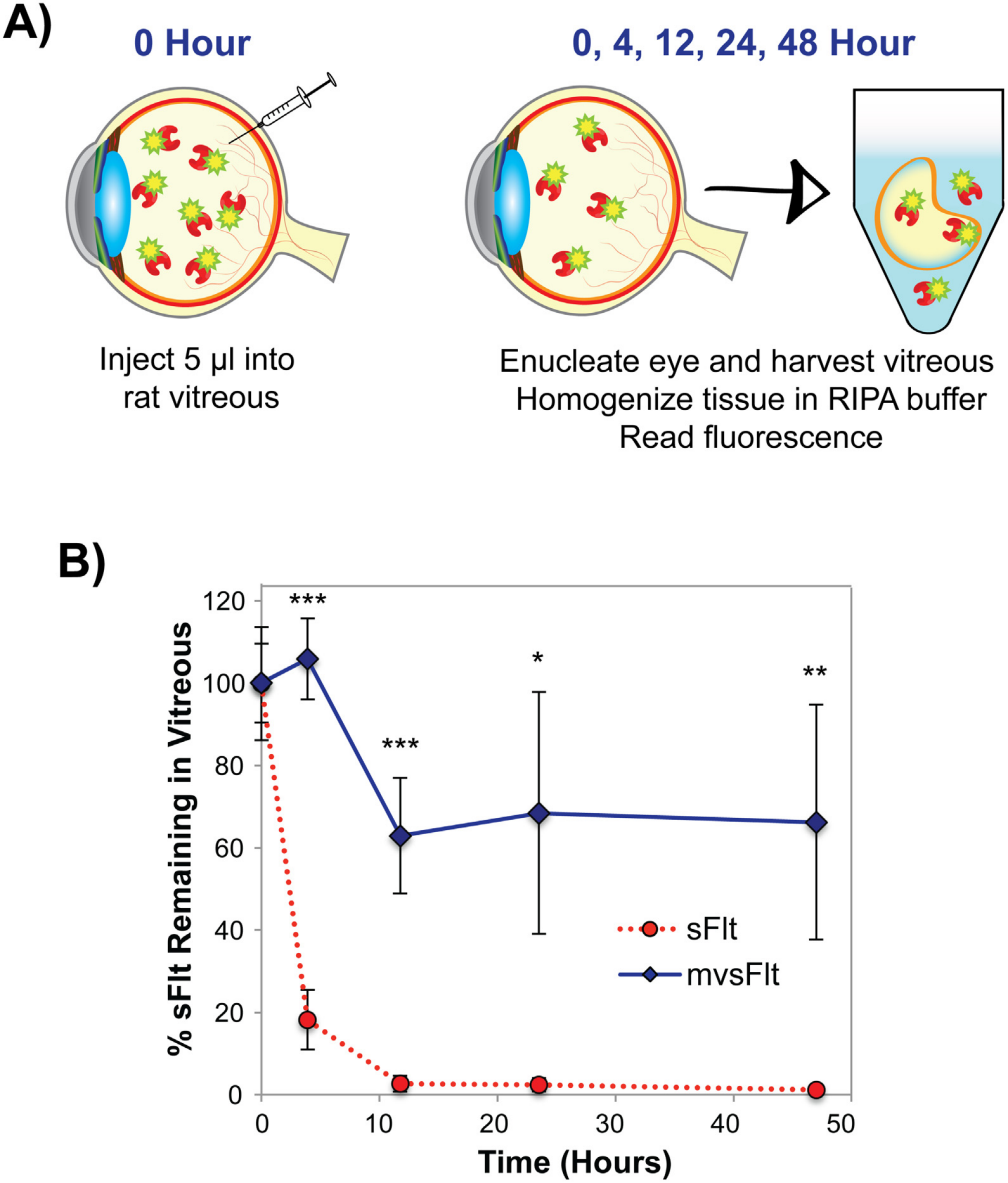
mvsFlt has longer residence time in the rat vitreous. A) Schematic depicting methods used to determine the half-life of fluorescently tagged sFlt and mvsFlt in the rat vitreous. The vitreous was injected with 5μl of Alexa Fluor 488-tagged sFlt or mvsFlt. After 0, 4, 12, 24, and 48 hours, the rats were sacrificed and their eyes were enucleated and frozen for analysis. The vitreous was then removed, immersed in RIPA buffer and homogenized for subsequent fluorescence measurements. B) Conjugation to HyA significantly improves residence time of sFlt in the vitreous after 48 hours in comparison to sFlt. Results are expressed as mean ±SD (*p<0.05, **p<0.01, ***p<0.001). * indicates a difference between the mvsFlt and sFlt at the given time point. Two-way ANOVA gives p-value ***<0.001.

We initially confirmed that the vitreous of Brown Norway rats could be used to determine intravitreal residence time of different sized molecules using fluorescently tagged dextrans of varying sizes (S1 Fig). Differences in residence time between sFlt and mvsFlt were immediately apparent beginning at 4 hours where only 18.2±7.3% of sFlt remained compared to 105.8±9.8% of mvsFlt (Fig 3). By 12 hours, only 2.6±1.9% of sFlt remained detectable compared to 62.9±14.1% of mvsFlt. By 2 days post injection, sFlt was almost undetectable (1.2±0.5%) whereas 66.2±28.6% of mvsFlt remained in the vitreous. The half-life of sFlt in the vitreous was calculated using Eqs 1 and 2 to be 3.3 hours compared to 35 hours for mvsFlt.

### 3.3 mvsFlt is a more potent inhibitor of retinal neovascularization

**Fig 4:**
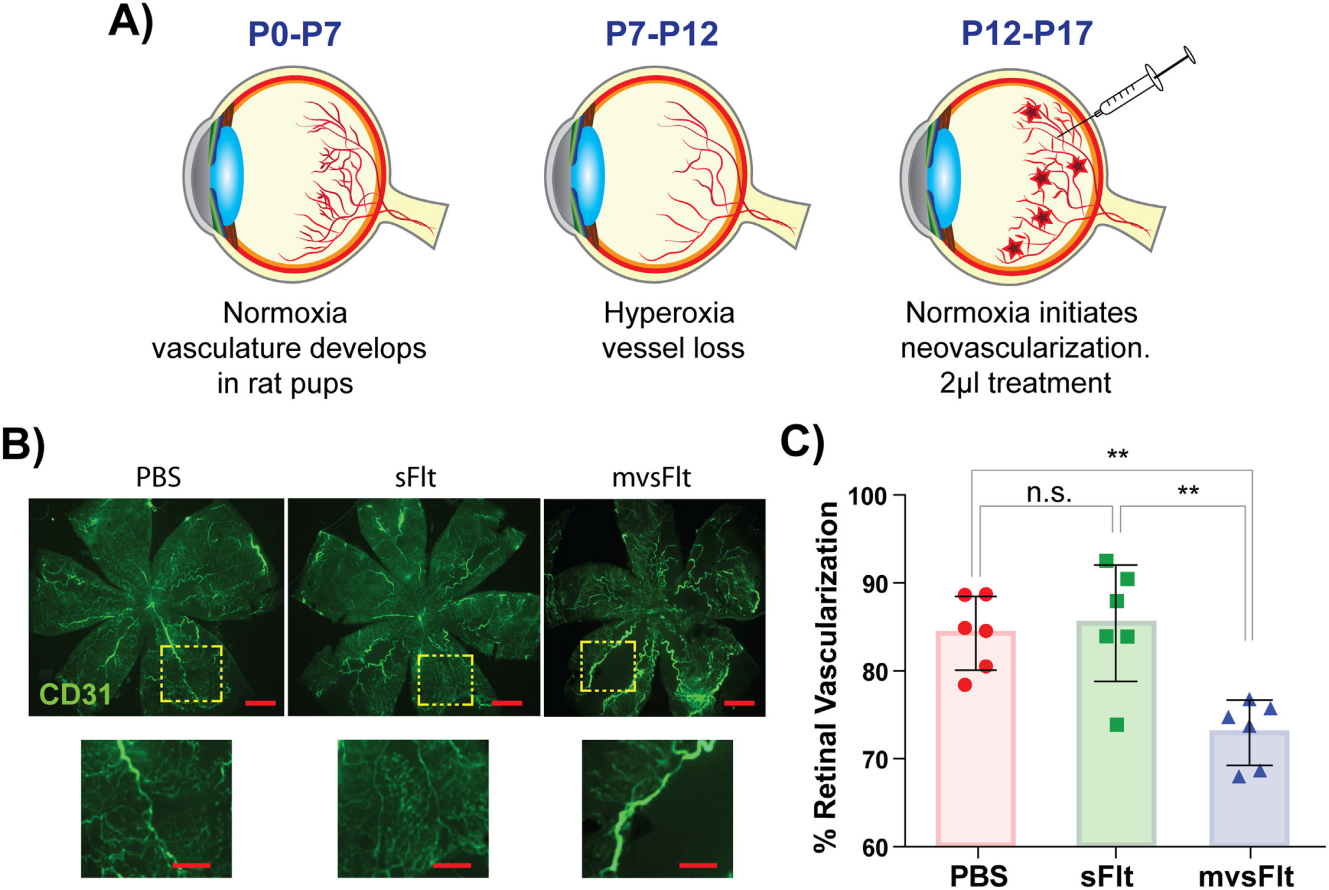
mvsFlt inhibits retinal angiogenesis. A) Schematic showing methods used for carrying out the OIR model. Newborn rat pups were housed in normoxic conditions (21% oxygen, room air) from post-natal day (P) 0–7 to allow for normal retinal vasculature development and then transferred to hyperoxic conditions from P7-P12, which induces vessel pruning. At P13, the pups are transferred back into normoxic conditions and treated with 2 μl of PBS, sFlt or mvsFlt and sacrificed at P17. B) Representative images of retinas treated with PBS, sFlt and mvsFlt. Prior to sacrifice, rat pups were injected with fluorescently-tagged 2 MDa dextran to visualize vessel coverage. Scale bar corresponds to 250 μm. Dashed boxes magnify that portion of tissue (scale bar corresponds to 100 μm). C) Quantified retinal vascularization after 5 days of treatment. Percent retinal vascularization was calculated by comparing the area of vascularization to the total retinal area in the image. One-way ANOVA gives p-value***<0.001 (n.s.-not significant; **p<0.01).

We next used an OIR model of retinal angiogenesis to examine the effect of HyA conjugation with sFlt on the inhibition of retinal angiogenesis. This short-term model allowed us to also indirectly examine the effect of mvsFlt half-life on prolonged angiogenesis inhibition. Neovascular coverage was calculated by comparing the area of vascular coverage to total retinal area. After 5 days of treatment, retinal vascular coverage of PBS-injected eyes was 84.3±3.8% and retinas treated with intravitreal injections of sFlt were 85.4±6.1%. In contrast, retinas from rats treated with intravitreal injections of mvsFlt were significantly lower and had 72.9±3.4% retinal vascular coverage (Fig 4).

## Discussion

The overall goal of this study was to determine whether increasing sFlt molecular size through conjugation to HyA would increase drug half-life in the vitreous and to investigate the ability of the multivalent sFlt bioconjugate to inhibit *in vivo* angiogenesis. In contrast to other drugs that are currently used for treating diabetic retinopathy that suffer from short half-lives, we have developed large macromolecules of multivalent protein bioconjugates that maintain their ability in inhibiting VEGF-driven angiogenesis *in vivo* and show significantly longer residence time in the vitreous.

Previously, we described a chemical scheme for creating multivalent protein bioconjugates of HyA and the anti-VEGF molecule, sFlt as shown in Fig 1 [12]. We were able to make several mvsFlt bioconjugates of varying molecular weights and protein valencies, all of which were equally able to inhibit VEGF in several *in vitro* assays of angiogenesis. We also demonstrated that conjugation of sFlt to HyA shielded the protein from protease degradation with a matrix metalloproteinase that is specific to sFlt, MMP-7 [18]. We created an *in vitro* model of the vitreous using crosslinked HyA hydrogels to examine the effect of HyA conjugation of sFlt diffusion. We found that low molecular weight mvsFlt (300 kDa), which had hydrodynamic diameter of 123.9 nm (±23.1 nm), had very similar diffusion profiles to sFlt whereas higher molecular weight mvsFlt (650 kDa and 1 MDa mvsFlt) diffused significantly slower. We chose to investigate the 650 kDa bioconjugate for its activity *in vivo*.

In this study, we used several different *in vivo* animal models for addressing the following three questions: 1) does the conjugation of sFlt to HyA affect the ability of sFlt to inhibit VEGF-driven angiogenesis; 2) does increasing the overall molecular size of sFlt lead to prolonged intravitreal half-life; and 3) does increasing intravitreal half-life prolong the inhibitory profile of sFlt. To answer our first question, we used a corneal angiogenesis assay as a method to easily visualize the effect of sFlt and mvsFlt on inhibition of angiogenesis. This assay is an excellent model for studying *in vivo* angiogenesis due to ease of vessel visualization on the physiologically avascular and transparent cornea, which rapidly vascularizes upon stimulation with a chemical burn or injury [19,20]. Results from the corneal angiogenesis assay indicated that subconjunctivally administered sFlt and mvsFlt equally inhibited VEGF-driven angiogenesis as shown in Fig 2. We did not anticipate a difference in angiogenesis inhibition between the conjugated and unconjugated protein due to the nature of clearance mechanisms in the cornea and the subconjunctival space [21]. Following injection of drugs into the subconjunctival space, drugs are rapidly cleared into systemic blood circulation via an extensive capillary network that is able to absorb large hydrophilic substances such as sFlt and mvsFlt due to the presence and abundance of large pores in the vascular epithelium [22,23]. We anticipated that the large pores in the vascular epithelium would clear both sFlt and mvsFlt at similar rates despite their significantly different sizes, which our results validated.

To address our second question, we examined the direct effect of HyA conjugation on the half-life of mvsFlt in the rat vitreous. We initially validated this model using fluorescently tagged dextrans and found that greater than 94% of the 40 kDa and 2 MDa dextrans were cleared within 48 hours (S1 Fig). Based on this data, we chose to examine the following 5 time points for examining the half-life of mvsFlt: 0, 4, 12, 24 and 48 hours. Significant differences in residence time were observed between sFlt and mvsFlt beginning at 4 hours and sustained until the end of the study at 48 hours as demonstrated in Fig 3. The half-lives of sFlt and mvsFlt were calculated according to Eqs 1 and 2 and were determined to be 3.3 and 35 hours, respectively. Conjugation to HyA therefore increased the half-life of sFlt by approximately 10-fold, an effect seldom seen in the literature, outside of implant technologies. In comparison, liposomal delivery of bevacizumab increased the drug half-life by 5 times [24] but this approach presents several challenges including complexity of synthesis, aggregation of liposomes and blurring of vision. PEGylation is another approach to improve the half-life of drugs. PEGylation of the aptamer in pegaptanib improved its half-life by 2.5 fold from 4.2 days [25] to 10±4 days [26].

The OIR model [27,28] enabled us to examine retinal neovascularization with the added advantage of also indirectly investigating the effect of conjugation on prolonged inhibition due to increased residence time mvsFlt conjugate in the vitreous, which helped us address our third and final question. In contrast to the cornea where conjugation does not show a significant advantage, the vitreous is a tissue where clearance mechanisms are highly dependent on molecular size [29,30]. Due to its increased hydrodynamic diameter, we anticipated that the mvsFlt conjugate would have a longer half-life in the vitreous and result in a prolonged duration of angiogenesis inhibition. The mvsFlt conjugate showed superior inhibition of retinal angiogenesis in this model in comparison to sFlt, which did not inhibit angiogenesis in comparison to the PBS control (Fig 4).

**Fig 5:**
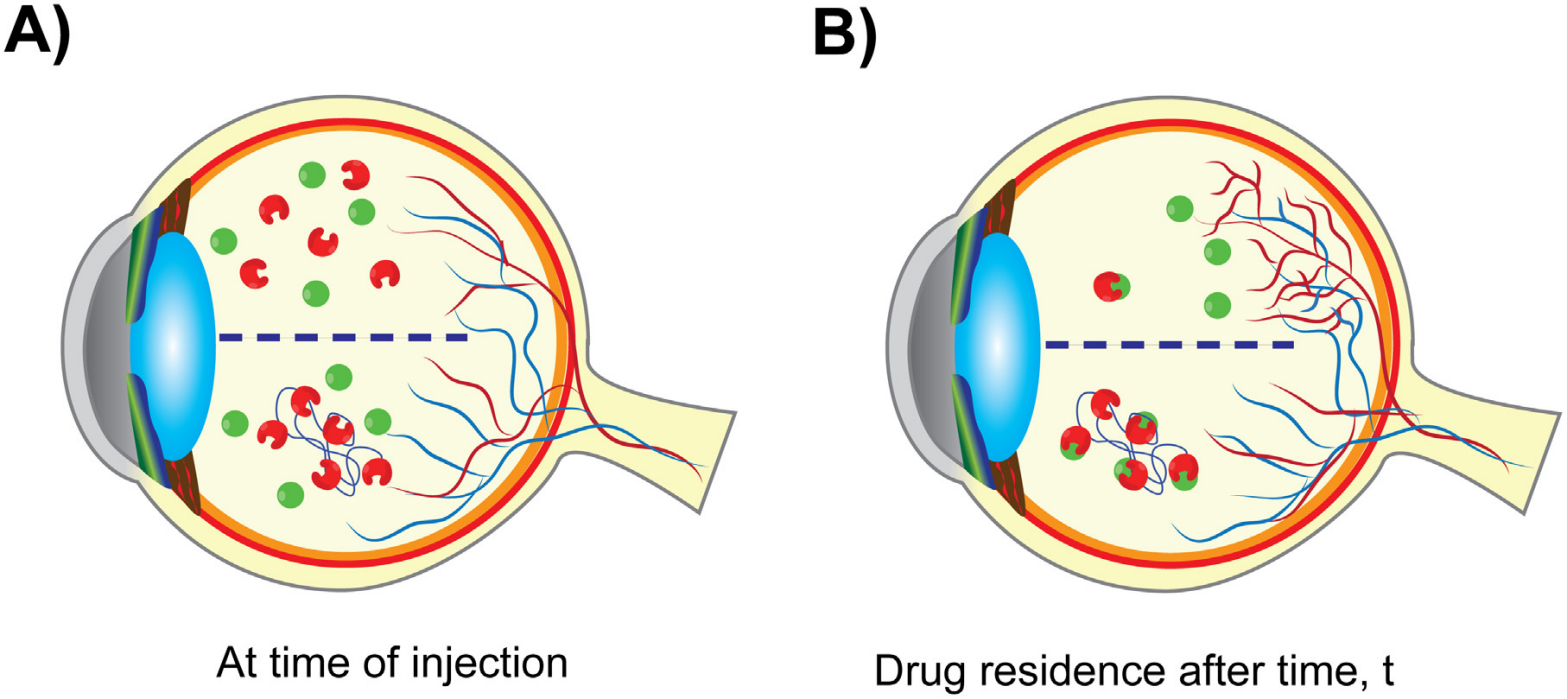
Schematic demonstrating the proposed mechanism of mvsFlt action. A) sFlt (red, unconjugated) and mvsFlt (red conjugated blue chain of HyA) are injected into a diabetic retina where there is a high concentration of VEGF (green circles). B) After a given time, t, the majority of the sFlt has been cleared from the vitreous and VEGF is thus able to induce blood vessel growth. mvsFlt has a longer residence time in the vitreous and is able to bind and inhibit VEGF over much longer periods of time, leading to prolonged inhibition of retinal angiogenesis.

We believe that this difference in inhibition of retinal angiogenesis is primarily due to clearance of the sFlt from the vitreous more rapidly in comparison to mvsFlt, resulting in ineffective angiogenesis inhibition. At the time of injection, both sFlt and mvsFlt have similar concentrations in the vitreous (Fig 5A). Over time, sFlt is small enough to be cleared from the vitreous leaving much lower concentrations of drug (Fig 5B, top). This allows for an increase in the intravitreal VEGF concentration, which induces angiogenesis. In contrast, mvsFlt has a much longer intravitreal residence time and is thus able to act as a sponge for VEGF over time (Fig 5B, bottom), inhibiting angiogenesis and maintaining the basal level of vascularization in the retina. The time over which this occurs is highly dependent on the animal model being used. Lucentis, which is similar in size to sFlt and is also a protein therapeutic, has an intravitreal half-life of 2.9 days in rabbits [31] and 2.6 days in rhesus monkeys [32]. The disparity in the half-lives of our compounds and those of therapeutics tested in rabbit and monkey models is most likely due to anatomical differences in ocular globe size and differences in the rate of vitreous turnover between species. We anticipate that the unconjugated sFlt would perform similarly to Lucentis in rabbit and monkey models whereas the mvsFlt would likely show significant improvement in half-life over currently used therapeutics based on our preliminary studies shown in Fig 3.

Comparable approaches have been taken to increasing intravitreal half-lives of anti-VEGF compounds such as the sFlt peptide presented by Oh et al. [33,34]. The approach involves the formation of micelles based on the hydrophobic nature of the sFlt-1 peptide and hydrophilicity of HyA. The use of these types of self-assembling nanoparticles in intravitreal applications has been documented in the past and often results in particle aggregation and blurring of vision due to particle instability [35,36]. The sFlt-1 protein fragment we developed is also significantly more active than the peptides described by Oh et al. We have performed similar VEGF_165_ ELISAs and our calculations indicate that the IC_50_ for our mvsFlt conjugates ranges between 50–150 pM, approximately 4 orders of magnitude more potent than the HyA-sFlt-1 peptides. Furthermore, due to the simplicity in the approach we outline, we are able to apply this technique to many anti-VEGF proteins including clinically approved Avastin and Lucentis. In our approach we use sFlt-1 as a model protein to demonstrate efficacy of a protein-based multivalent conjugate. We believe that similar results would be achieved with clinically approved protein-based therapeutics based on similarity in protein charge, size and activity.

The current approach to treating retinal neovascularization including diabetic retinopathy is carried out through intravitreal injection through the sclera. Once injected into the vitreous, drugs distribute through the vitreous in a charge and size dependent manner and are cleared from the vitreous through two main routes: anterior and posterior elimination. Small molecules are able to diffuse through the vitreous rapidly and have short half-lives whereas large molecules have retarded diffusion that results in longer half-lives. Large, hydrophilic molecules are able to passively diffuse through the vitreous, around the lens into the anterior chamber and are cleared through the Schlemm’s canal or the uveoscleral outflow pathway. Due to the large surface area of the retina, molecules capable of posterior clearance have significantly lower half-lives in comparison to those eliminated through the anterior route [21,37].

The development of drugs with longer residence times for patients with diabetic retinopathy has been in high demand with the increasing prevalence of diabetes in populations worldwide. Currently used drugs have short half-lives in the vitreous due to small molecular size and thus require patients to receive frequent intravitreal injections, leading to low patient compliance and worsening of the disease. Clinically approved drugs Avastin, Lucentis, and Eylea have half-lives of 7.4 [38], 6.7 [31,32], and 5 days [39,40], respectively and require patients to receive injections every 4–6 weeks to maintain effective levels of intravitreal drug [8]. Several different approaches have been taken to increase drug residence time in the vitreous for treating diseases of the posterior segment, each with its own advantages and drawbacks. Intravitreal implants have received much of the attention in intravitreal drug delivery over the last two decades and have primarily been used for corticosteroid delivery in posterior uveitis, diabetic macular edema (DME), and retinal vein occlusion. Implantable devices are able to deliver a constant supply of drug over several months to several years. Several types of implants have been developed such as Iluvien [41], Ozurdex [42], and Retisert [43] and vary in degradability, duration of effect, size, delivery method and composition. Despite being able to deliver drug over long periods of time, implants often require surgical implantation and invasive removal of the implant after the drug is depleted. Particulate systems such as liposomes and nanoparticles have also emerged as a drug delivery option for treating retinal diseases. Although drug delivery with particulate systems provides stability for the entrapped drug and is more straightforward requiring minimally invasive intravitreal injections, this approach is accompanied by several disadvantages including the formation of precipitate that blurs vision, increased production cost and heterogeneity in formulation due to aggregation of particles [36,44]. PEGylation has also been employed to increase intravitreal half-life of drugs and is the core technology behind Macugen, a PEGylated anti-VEGF aptamer (Bausch + Lomb). Although similar in theory to our approach, PEGylation uses a synthetic polymer for conjugation to a single biological molecule, which often results in significantly decreased drug bioactivity [45,46]. Furthermore, PEGylation on the Macugen aptamer did not result in a drug with significantly longer half-live compared to clinically available drugs, most likely due to the small size of polyethylene glycol chain that employed [47].

In contrast to these approaches, we employed a multivalent conjugate approach with HyA, a naturally occurring biopolymer found in high concentrations within the vitreous, and conjugated to it several molecules of sFlt, a potent anti-VEGF protein. HyA was chosen not only based on its natural ubiquity within the eye and body but also due to its exceptionally low turnover rate within the vitreous, 30 days [48]. We have shown that conjugation of sFlt to HyA does not negatively impact the ability of sFlt to bind and inhibit VEGF *in vitro* [12] or reduce its anti-angiogenic potency *in vivo* (Fig 2), effects that accompany the other conjugation technology, protein PEGylation. Furthermore, we demonstrated that conjugation to HyA significantly improved the residence time of sFlt in the vitreous of the eye (Fig 3), which resulted in prolonged inhibition of retinal angiogenesis (Fig 4). Given these promising results, we anticipate that the future clinical use of multivalent conjugates of sFlt may significantly increase the intravitreal drug residence time and inhibit retinal angiogenesis over a longer period of time. Due to the ease of synthesis and tunability in this approach, we believe that our conjugation technology could be instrumental in improving the half-life of drugs in the human eye not only for treating diabetic retinopathy but other diseases as well.

## Supporting Information

S1 Fig Higher molecular weight dextran displays longer residence time *in vivo*. A) Validation experiment demonstrating the effect of size on the retention of fluorescently tagged dextrans. The 2 MDa dextran (solid line) has significantly improved residence time over the 40 kDa (dashed line) over 48 hours. The half-life of the 40 kDa and the 2 MDa dextrans is 3.2 and 5 hours, respectively. * indicates a difference between the 40 kDa and 2 MDa dextran at the given time point. Two-way ANOVA gives p-value* <0.05 (** corresponds to a P-value less than 0.01).
(TIF)
