# Supplementary figures and images for: sFlt Multivalent Conjugates Inhibit Angiogenesis and Improve Half-Life In Vivo
Source: PLoS One. 2016 Jun 3;11(6):e0155990. doi: 10.1371/journal.pone.0155990 (PMC4892585; doi:10.1371/journal.pone.0155990)

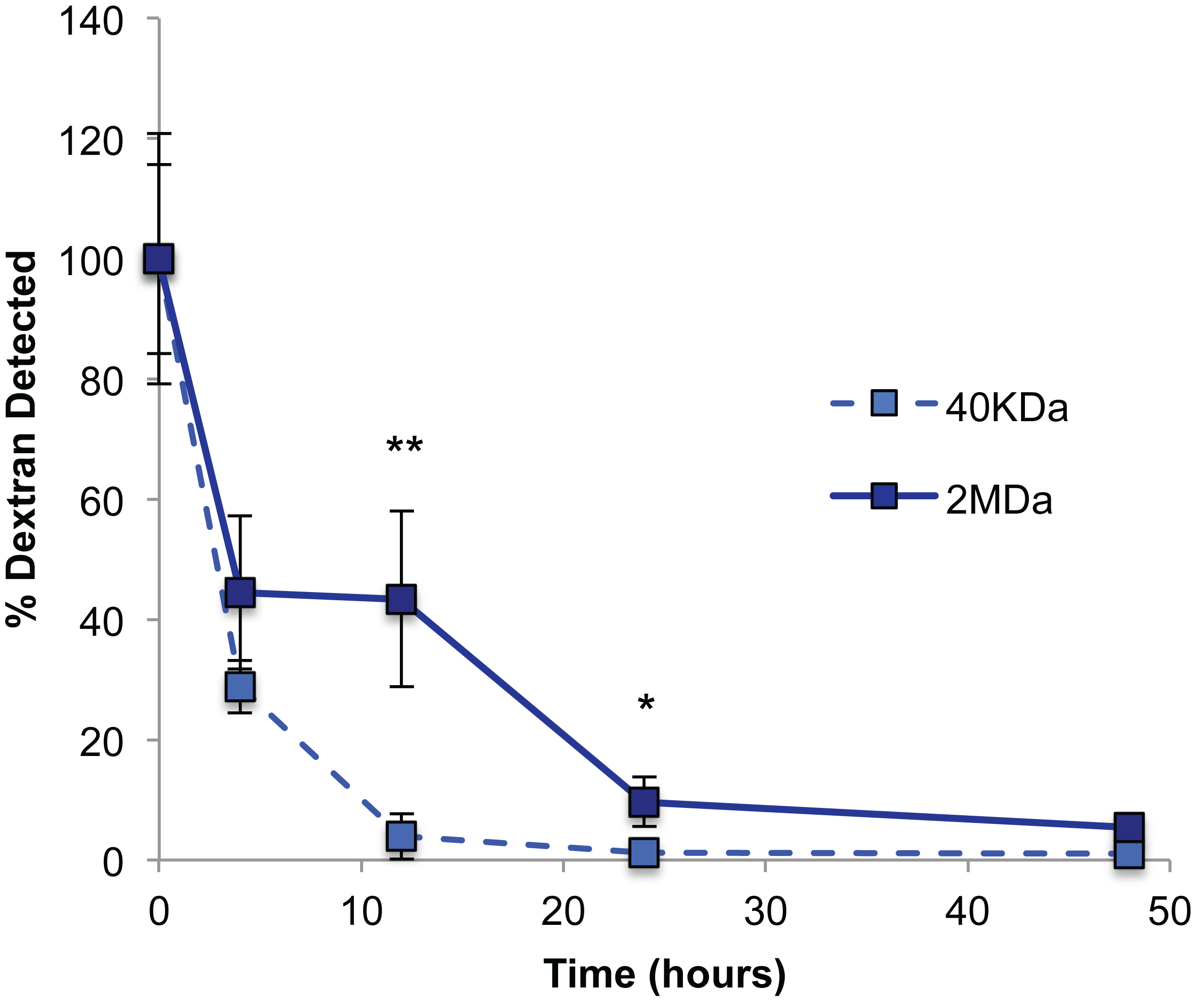

Supplement: S1 Fig — A) Validation experiment demonstrating the effect of size on the retention of fluorescently tagged dextrans. The 2 MDa dextran (solid line) has significantly improved residence time over the 40 kDa (dashed line) over 48 hours. The half-life of the 40 kDa and the 2 MDa dextrans is 3.2 and 5 hours, respectively. * indicates a difference between the 40 kDa and 2 MDa dextran at the given time point. Two-way ANOVA gives p-value* <0.05 (** corresponds to a P-value less than 0.01). (TIF) [file pone.0155990.s001.tif]
